# GALA: an international multicentre randomised trial comparing general anaesthesia versus local anaesthesia for carotid surgery

**DOI:** 10.1186/1745-6215-9-28

**Published:** 2008-05-21

**Authors:** Michael J Gough, Andrew Bodenham, Michael Horrocks, Bridget Colam, Steff C Lewis, Peter M Rothwell, Adrian P Banning, David Torgerson, Moira Gough, Demosthenes Dellagrammaticas, Anne Leigh-Brown, Christos Liapis, Charles Warlow

**Affiliations:** 1 Vascular Surgical Unit, Leeds General Infirmary, Great George Street, Leeds LS1 3EX, UK; 2 Department of Anaesthesia, Leeds General Infirmary, Great George Street, Leeds LS1 3EX, UK; 3 University Department of Surgery, Royal United Hospital Trust, Combe Park, Bath, BA1 3NG, UK; 4 Neurosciences Trials Unit, Division of Clinical Neurosciences, Edinburgh University, Western General Hospital, Crewe Road, Edinburgh EH4 2XU, UK; 5 Department of Clinical Neurology, Radcliffe Infirmary, Woodstock Road, Oxford OX2 6HE, UK; 6 Department of Cardiology, Radcliffe Infirmary, Woodstock Road, Oxford OX2 6HE, UK; 7 Central for Health Economics, University of York, Heslington, York YO1 5DD, UK; 8 Data Intelligence Group, Information Services Division, NHS National Services Scotland, Gyle Square, Edinburgh EH12 9ED, UK; 9 Department of Vascular Surgery, Athens University Medical School, Laiko General Hospital, 16 Sevastoupoleos Street, 115 26 Athens, Greece

## Abstract

**Background:**

Patients who have severe narrowing at or near the origin of the internal carotid artery as a result of atherosclerosis have a high risk of ischaemic stroke ipsilateral to the arterial lesion. Previous trials have shown that carotid endarterectomy improves long-term outcomes, particularly when performed soon after a prior transient ischaemic attack or mild ischaemic stroke. However, complications may occur during or soon after surgery, the most serious of which is stroke, which can be fatal. It has been suggested that performing the operation under local anaesthesia, rather than general anaesthesia, may be safer. Therefore, a prospective, randomised trial of local versus general anaesthesia for carotid endarterectomy was proposed to determine whether type of anaesthesia influences peri-operative morbidity and mortality, quality of life and longer term outcome in terms of stroke-free survival.

**Methods/design:**

A two-arm, parallel group, multicentre randomised controlled trial with a recruitment target of 5000 patients. For entry into the study, in the opinion of the responsible clinician, the patient requiring an endarterectomy must be suitable for either local or general anaesthesia, and have no clear indication for either type. All patients with symptomatic or asymptomatic internal carotid stenosis for whom open surgery is advised are eligible. There is no upper age limit. Exclusion criteria are: no informed consent; definite preference for local or general anaesthetic by the clinician or patient; patient unlikely to be able to co-operate with awake testing during local anaesthesia; patient requiring simultaneous bilateral carotid endarterectomy; carotid endarterectomy combined with another operation such as coronary bypass surgery; and, the patient has been randomised into the trial previously. Patients are randomised to local or general anaesthesia by the central trial office. The primary outcome is the proportion of patients alive, stroke free (including retinal infarction) and without myocardial infarction 30 days post-surgery. Secondary outcomes include the proportion of patients alive and stroke free at one year; health related quality of life at 30 days; surgical adverse events, re-operation and re-admission rates; the relative cost of the two methods of anaesthesia; length of stay and intensive and high dependency bed occupancy.

**Trial registration:**

Current Controlled Trials ISRCTN00525237

## Background

Patients who have severe narrowing at or near the origin of the internal carotid artery as a result of atherosclerosis have a high risk of ischaemic stroke ipsilateral to the arterial lesion. Carotid endarterectomy to remove the lesion is often advised, particularly soon after a prior transient ischaemic attack or mild ischaemic stroke. It is less often appropriate if the arterial lesion is asymptomatic. The operation involves surgical exposure of the carotid artery followed by removal of the atheromatous plaque that is causing narrowing of the artery.

A number of complications may occur during or soon after carotid endarterectomy, the most serious of which is a stroke (5–7% of cases), which can be fatal. Nonetheless, two large multicentre trials have confirmed the advantage of surgery in the long-term prevention of ischaemic stroke ipsilateral to the operation, for symptomatic patients [1] and there may also be some advantages for asymptomatic stenosis patients [2]. However, the complications of surgery cannot be dismissed and anything that might reduce their frequency would be very important. One option is to do the operation not under general anaesthesia which is the most usual practice, but under local or regional anaesthesia because it may be safer. Both techniques have been in widespread use ever since the operation was introduced in the late 1950s.

In a meta-analysis [3] of 41 non-randomised studies (25,622 patients) and seven randomised studies (554 patients), carotid endarterectomy under local anaesthesia was associated with about a 40% reduction in the relative odds of peri-operative stroke or death. Whilst this is of considerable interest, the findings must be viewed with caution. Almost all the data came from the non-randomised studies, which are susceptible to publication bias, patient selection bias and the unequal distribution of risk factors at baseline. Many studies were retrospective and included non-consecutive patients. Furthermore, the distribution of patients with asymptomatic stenosis (low risk), and variations in surgical technique and antiplatelet therapy were biased in favour of local anaesthesia. As a result, this review can only assist in the generation of a hypothesis for further examination.

The potential benefits of local anaesthesia in a wider range of surgical procedures is supported by an overview of randomised trials of spinal and epidural anaesthesia versus general anaesthesia; mortality was reduced by about one-third in patients allocated to neuraxial block [4]. Complications of surgery were also reduced, but again sample sizes were small and many different types of patient and procedure were combined in the analysis. In addition, a number of studies were confounded by the use of regional anaesthesia as an adjunct to general anaesthesia.

Therefore, a prospective, randomised trial of local versus general anaesthesia for carotid endarterectomy is proposed to determine whether the type of anaesthesia does indeed influence peri-operative morbidity and mortality, quality of life and long term outcome in terms of stroke-free survival. If so, this finding might be generalisable to other surgical procedures, or at least encourage further randomised controlled trials.

## Design and methods

GALA is a two-arm, parallel group, multicentre randomised controlled trial. Patients will be randomised to either surgery under local anaesthesia (superficial and deep cervical plexus nerve block) or general anaesthesia. Ethical approval was obtained from the Northern and Yorkshire Multicentre Research Ethics Committee for the pilot phase in August 1998 and for the main phase in April 2003.

### Primary outcome

The primary outcome is the proportion of patients alive and stroke-free (including retinal infarction) and without myocardial infarction at 30 days after surgery, or 30 days after randomisation for those few patients for whom surgery was scheduled but was never carried out.

### Setting

Each trial centre must be experienced in carotid surgery (at least 15 procedures per consultant surgeon per annum) and have local research ethics approval. Each centre will provide the Trials Office with the number of patients operated on in the previous year, and of them, how many had local anaesthesia. Preoperative assessment of carotid disease and the technical aspects of surgery will be whatever is the standard practice in trial centres.

The issue of surgical procedures performed by trainees is difficult. Ideally either all surgery performed within the study should be undertaken by a trained consultant vascular surgeon, or equal numbers of patients in each arm of the study should be operated on by trainees under supervision. Although the latter is likely to better match current clinical practice, there may be some difficulty in ensuring that this actually happens. Individual centres will therefore be asked to monitor this carefully if it is their intention to allow trainees to operate on study patients. A further check will be made in the Trials Office, and any necessary feedback provided to the participating centres to ensure as far as possible that similar proportions of patients in each treatment arm are operated on by consultants and trainees. The alternative, that only non-randomised patients provide the focus for training, is not ethically acceptable. The same issue applies to trainee anaesthetists.

### Inclusion criteria

Any patient requiring carotid endarterectomy, who is considered suitable for either local or general anaesthesia. All patients with either symptomatic or asymptomatic internal carotid stenosis for whom surgery is advised are eligible. There is no upper age limit. Fully informed consent is required from all patients.

### Exclusion criteria

• Unable to obtain informed consent.

• Patient unlikely to be able to co-operate with awake testing during local anaesthesia.

• Patient considered unfit for general anaesthesia.

• Patient considered unfit for local anaesthesia.

• Patient requiring simultaneous bilateral carotid endarterectomy.

• Carotid endarterectomy combined with another operative procedure, e.g. coronary artery bypass surgery

• Patient has been randomised into the trial previously

### Intervention

Some may believe that many characteristics of the study groups should be strictly standardised, including: the exact indications for surgery, diagnostic methods, anaesthetic techniques, surgical techniques (indications for, and the use of shunts; heparin dose; patching etc), intra-operative monitoring, post-operative assessment, antiplatelet therapy, and the proportion of procedures undertaken by surgical trainees. This is impractical in the context of a multicentre international study and unnecessary. With the exception of the following, we suggest that current practices and protocols within trial centres should continue unchanged (of course, data on all of the above will be collected): 1. The issue of trainees discussed above; 2. Shunts should generally only be used in the local anaesthesia patients when awake neurological testing indicates a need, this being an inevitable and central part of the protocol for the local anaesthesia approach.

As a result of this pragmatic approach the results will be more generalisable since we will be comparing the outcome of surgery under either local or general anaesthesia in real clinical settings where procedures are not necessarily all performed in exactly the same way between centres, or even between surgeons in the same centre.

As far as the anaesthetic techniques employed in this study are concerned a pragmatic approach to standardisation will also be taken. In other words each centre will continue with their standard anaesthetic protocol. Strict guidelines on anaesthetic management are unnecessary given that each surgical procedure is a 'package' of anaesthetic and surgical care. In general terms the anaesthetic protocols shown in table 1 should be used.

**Table 1:** Anaesthesia protocols

**General anaesthesia**	**Local anaesthesia**
Premedication: benzodiazepine or none	Premedication: benzodiazepine or none
Intravenous access + arterial line under local anaesthetic	Intravenous access + arterial line under local anaesthetic
Intravenous induction + opiate analgesia	Judicious conscious sedation (e.g. benzodiazepine/opiate)
Muscle relaxant	Deep and superficial cervical plexus block + local infiltration (e.g. with bupivacaine)
Tracheal intubation, ventilation to normocapnia	Intra-operative top-up of local anaesthetic if required (lignocaine)
Maintain systemic blood pressure to pre-operative levels with intravenous fluids/cardioactive drugs if required	Maintain systemic blood pressures to pre-operative levels with intravenous fluids/cardioactive drugs if required
Reversal of anaesthetic and extubation	O_2 _as necessary by nasal cannulae/mask, in particular during cross-clamping of carotid vessels
Oxygen overnight by nasal cannulae/mask	

Each trial centre will continue with their standard anaesthesia protocol. Strict guidelines on anaesthetic management are unnecessary given that each surgical procedure is a 'package' of anaesthetic and surgical care. In general terms the anaesthesia protocols in this table should be used.

In summary, the study will allow participating centres to continue to undertake carotid endarterectomy in the same way as they currently practice, with no specific constraints or restrictions.

The technique of superficial and deep cervical nerve block is familiar to many anaesthetists, particularly those who work in pain clinics. For those centres that are not familiar with this technique two alternatives are available. Firstly, any anaesthetist/surgeon can visit the General Infirmary at Leeds to observe one or more procedures. Secondly, a video of both the administration of local anaesthesia and the 'in-theatre' set-up for surgery is available. Before randomising any patients, each centre is expected to have done at least five procedures under local anaesthesia.

### Sample size

The study requires 5,000 patients to demonstrate a clinically worthwhile difference between the two anaesthesia techniques in the primary outcome – based on a predicted one third reduction in the risk of stroke (including retinal infarction), myocardial infarction or death within 30 days of surgery, when it is performed under local as compared with general anaesthesia (from 7.5% to 5%, a more conservative treatment effect than suggested by the meta-analysis). This sample will provide a more than 90% chance of detecting this treatment difference at the 5% level.

To recruit 5,000 patients by the end of 2007 we will need at least 100 centres in the UK, mainland Europe, and elsewhere if particularly interested.

### Randomisation

Randomisation is stratified by centre and uses balanced blocks of variable size. The randomised treatment allocation is provided centrally by the Trials Office in Edinburgh. Balance between the two groups is checked periodically by the trial statistician. Baseline data are collected by the local collaborator, written on a randomisation note pad [see Additional file 1], and faxed to the Trials Office who then fax back the randomised treatment allocation.

Because all outcomes will be counted and analysed from the moment of randomisation, it is important that randomisation itself occurs fairly close to the day of surgery – perhaps the day before, or a few days before at most. To allow patients to think about consent to randomisation, more time for reflection may well be required, so the consent process might be usefully carried out during the clinic appointment when surgery itself is discussed. So the general rule is 'consent early, but randomise late'. Exactly how this process gets done will depend on local factors in each centre.

From the moment of randomisation the patient is considered to be in the trial and accounted for in the analysis whether or not they received the allocated treatment intervention, or even surgery at all, i.e. the analysis is on an intention-to-treat basis unless otherwise stated.

### Data collection at baseline

Factors which might predict surgical risk and long-term vascular events are recorded and the data checked in the Trials Office for completeness and consistency. Data to be collected include: demographic information, details of the cerebrovascular event(s), vascular risk factors, diagnostic procedures and findings (brain and arterial imaging), indications for surgery and illness severity grading.

### Data collection at 7 days post-operatively, hospital discharge or death

Data collection forms [see Additional file 2] are supplied to each centre and the completed forms posted or faxed back to the Trials Office. Data to be collected comprise details of the surgery and anaesthetic, and outcomes. The surgical data collected include the date the surgical procedure was carried out, reasons for not having surgery at all or for surgery to be abandoned before completion, details of the anaesthesia used, reasons for any cross over from allocated treatment, skin incision to skin closure duration of surgery. We also collect the grade of the surgeon and anaesthetist (consultant or trainee), recovery room/intensive care/high dependency unit occupancy, and length of stay. The outcome data collected include stroke (including retinal infarction), with severity determined by modified Rankin scale six months after onset by post to the patient's general practitioner (or other appropriate physician); myocardial infarction; death (and cause); transient ischaemic attacks; other complications e.g. re-operation, neck haematoma, respiratory or urinary problems, cranial nerve palsy etc.

### Data collection at one month

Data collection forms [see Additional file 3] are supplied to each centre and the completed forms are posted or faxed to the Trials Office. The following data are collected by an independent stroke physician or neurologist as 'blind' as possible to the type of anaesthesia that the patient received; stroke (including retinal infarction), severity determined by modified Rankin scale six months after onset by post to the patient's general practitioner (or other appropriate physician); myocardial infarction; death (and cause); transient ischaemic attacks; and other complications, respiratory problems, or cranial nerve palsy.

Separate forms are also sent by post direct to all UK patients from the Trials Office to collect quality of life data (SF36 and EuroQol). Non-UK patients do not receive this questionnaire.

### Follow-up at one year

Follow-up at one year will be preceded by contacting each patient's General Practitioner or other relevant physician to ensure that the patient is still alive and suitable for a follow-up questionnaire on paper. The following data will be collected: stroke (including retinal infarction), severity determined by modified Rankin scale six months after onset by post to the patient's general practitioner (or other appropriate physician); death (and cause); myocardial infarction; whether the patient is currently smoking; any medications for high blood pressure, lipid lowering and antithrombotic (to ensure there is no systematic bias in the use of co-interventions which could affect stroke risk).

In non-UK countries different arrangements will be made for annual follow-up, probably telephone contact by a stroke physician from one of the country trial centres.

### Checking and auditing primary outcomes

Details of any stroke (including retinal infarction), myocardial infarction and death are collected by the Trials Office and a clinician (as blind as possible to treatment allocation) prepares a clinical summary. This summary is then sent to an independent neurologist or cardiologist for audit, completely blinded to treatment allocation. Information is requested from hospital notes, death certificates and general practitioner records where appropriate. Enquiry is also made to ensure that any reported transient ischaemic attacks are not in fact minor strokes. Stroke is defined by the usual World Health Organisation criteria using a 24-hour cut off from transient ischaemic attacks. Myocardial infarction and other coronary events are defined on the basis of the history, electrocardiogram and cardiac enzymes.

### Data analysis

Data analysis will be performed by the Principal Investigators in collaboration with the trial statistician at the Neurosciences Trials Unit, Edinburgh University. Trial data will be presented according to the CONSORT guidelines [5]. All analyses unless otherwise stated will be by intention-to-treat and will compare all patients allocated to general anaesthesia with all those allocated to local anaesthesia. For the primary outcome, we will present the relative and absolute differences in the proportion of patients alive and stroke-free (including retinal infarction) and without myocardial infarction at 30 days after surgery, or 30 days after randomisation for those few patients for whom surgery was scheduled but was never carried out. A variety of other secondary analyses (with due allowance for their exploratory nature) will be performed to compare: survival to 30 days post-surgery, other peri-operative adverse events; survival analysis of stroke, myocardial infarction and death in the longer term at one year; length of stay in recovery, high dependency unit, intensive therapy unit and overall; and health related quality-of-life at about 30 days post-surgery.

### Health economic analysis

Our systematic review suggested that patients having surgery under local anaesthesia spend less time in intensive care and in hospital. This is not a robust finding since only six studies gave relevant data and only two indicated the duration of intensive care. In GALA we will collect data on length of stay in various parts of the hospital, and the number and estimated costs of strokes during follow-up, because if local anaesthesia reduces the number of strokes this will be an important cost saving. As well as estimating the differential costs of the two approaches, we will, if local anaesthesia is shown to be clinically superior, undertake a modelling study to estimate the likely cost utility of carotid endarterectomy once the clinical risk benefit ratio has been altered. If local anaesthesia were shown to be superior it is likely to encourage surgeons to operate on lower risk (without surgery) patients and higher risk (with surgery) patients than at present. We wish to see if such a clinical policy is cost effective.

### A priori subgroups

We will present the relative and absolute differences in the proportion of patients alive and stroke-free without myocardial infarction at 30 days post surgery subdivided by: low baseline risk of surgical stroke or death versus high baseline risk of surgical stroke or death using a previously published model [6]; by age (i.e. young versus old); and by contralateral versus no contralateral carotid occlusion.

### Sensitivity analysis

Although unlikely, it is conceivable that surgeons beginning to operate under an unfamiliar anaesthetic regime (more likely local than general) may have more operative problems than usual. We will be discussing how to analyse the trial without the 'learning curve' with colleagues and the Data Monitoring Committee.

### Generalisability of the trial results

The results of this trial can be applied to similar patients in the future. It is, therefore, very important to describe in detail the characteristics of the patients randomised, as we will do. Inevitably, there will be some eligible patients who, for some reason, do not get randomised. However, our inclusion criteria are broad, and we envisage that across all centres, a very wide variety of patients will be randomised. We will collect the numbers of operated patients in each trial centre to gain some idea of the overall experience of the centre and what proportion of their patients enter the trial.

### Organisation

The Steering Committee will usually meet every six months to review the progress of the trial, including the overall complication rates of surgery. However, apart from the trial statistician, all the members of the committee will remain blinded to any outcomes and other important results divided by treatment allocation.

All members of the Data Monitoring Committee will be totally independent of the trial. The Data Monitoring Committee will regularly review the recruitment into the trial, trial discipline, the baseline data and the main outcomes. They will advise the Steering Committee on the adequacy of recruitment, the balance of the two treatment arms, and that there is no accumulating evidence of harm to one arm or the other. In this task they may request whatever unblinded analyses they wish from the trial statistician, and at whatever interval they wish. The Committee may also wish to consider any new information arising from other studies. All unblinded analyses are supplied to the Data Monitoring Committee in confidence.

The Data Monitoring Committee will advise the chairman of the Steering Committee if, in their view, the randomised comparisons have provided both: (i) 'proof beyond reasonable doubt' that for all, or some patients, one method of anaesthesia is clearly indicated or clearly contra-indicated; and (ii) evidence that might reasonably be expected to materially influence future patient management. Appropriate criteria of proof beyond reasonable doubt cannot be specified precisely, but we imagine the DMC will work to the well established guide that a difference of at least 3 standard errors in an interim analysis of the primary outcome events (between patients allocated local anaesthesia and patients allocated general anaesthesia) may be needed to justify halting or modifying the study before the planned completion of recruitment. This criterion has the practical advantage that the exact number of interim analyses is of little importance, and so no fixed schedule is proposed.

## Competing interests

The authors declare that they have no competing interests.

## Authors' contributions

All authors were involved in the conception and design of the trial, and the writing, revision and final approval of the final manuscript.

## Supplementary Material

Additional file 1 Randomisation notepad. The GALA trial randomisation data collection form. Click here for file

Additional file 2 Hospital discharge or 7 day post-surgery follow-up form. The GALA trial post-surgery data collection form. Click here for file

Additional file 3 One month post-surgery follow-up form. The GALA trial one-month post-surgery data collection form, for use at examination by independent neurologist. Click here for file
