# Supplementary material for: GALA: an international multicentre randomised trial comparing general anaesthesia versus local anaesthesia for carotid surgery
Source: Trials. 2008 May 21;9:28. doi: 10.1186/1745-6215-9-28 (PMC2413207; doi:10.1186/1745-6215-9-28)
Supplement: Additional file 1 — Randomisation notepad. The GALA trial randomisation data collection form. [file 1745-6215-9-28-S1.pdf]

GALA GENERAL ANAESTHESIA vs LOCAL ANAESTHESIA FOR CAROTID SURGERY
RANDOMISATION NOTEPAD

Patients are eligible if:

- require carotid endarterectomy for either symptomatic or asymptomatic carotid stenosis
- clinician is uncertain whether to recommend general or local anaesthetic
- unilateral carotid endarterectomy is the only operation being done
- have given his or her informed consent to randomisation

Patients are not eligible if:

- clinician is certain about whether to recommend local or general anaesthetic
- require bilateral simultaneous endarterectomy
- patient has previously been randomised in this trial
- unable to co-operate with awake testing

HOSPITAL CODE NUMBER: |__|__|__| or Hospital name |______________________________|

PATIENT DETAILS:

Family name: |____|

First names: |______________________| Hospital number |______________|

Date of birth: dd |____| / mm |____| / yyyy |____| Male 1 Female 2

Patient has given informed consent? [ ] **YES (MUST be answered 'Yes' before patient can be randomised)**

Patient able to co-operate with awake testing? [ ] **YES (MUST be answered 'Yes' before patient can be randomised)**

DATE FOR OPERATION *(dd/mm/yyyy)* ____/____/____ **Actual date / Estimated date** (delete as appropriate)

NAME OF RANDOMISING DOCTOR |________________________________| PLEASE PRINT CLEARLY

TODAY'S DATE (*dd/mm/yyyy*) ____/____/______

CEREBROVASCULAR EVENTS AT ANY PREVIOUS TIME

Please tick either Yes or No for each question and enter all relevant dates and numbers of events

		YES	NO		IF YES:	Date (dd/mm/yy) of most recent event
1.	Hemispheric stroke (carotid distribution)	[ ]	[ ]	➔	Right brain	
					Left brain	
2.	Hemispheric TIA (carotid distribution)	[ ]	[ ]	➔	Right brain	
					Left brain	
3.	Vertebrobasilar stroke	[ ]	[ ]	➔	**IF YES:**	
4.	Vertebrobasilar TIA	[ ]	[ ]	➔	**IF YES:**	
5.	Retinal infarct	[ ]	[ ]	➔	Right eye	
					Left eye	
6.	Amaurosis fugax (transient monocular visual loss)	[ ]	[ ]	➔	Right eye	
					Left eye	
7.	Carotid artery for surgery is asymptomatic*	[ ]	[ ]			

*** Asymptomatic = the CAROTID artery for surgery has NEVER ever had any symptoms**

*** Asymptomatic = if the patient has ONLY had vertebrobasilar stroke or TIA. (This artery by definition is asymptomatic.)**

8. Infarct visible on CT or MRI? *(tick all that apply)*

None visible	[ ]
Yes, on Right Side of brain	[ ]
Yes, on Left Side of brain	[ ]
**or** CT/MR not done	[ ]

THE CAROTID ARTERIES (The ECST method for measuring % carotid stenosis is shown in your trial information pack**. Use ECST criteria, or equivalent**)

(tick all that apply)

9. Method of assessment of stenosis:
   - Ultrasound ☐
   - Catheter Angiography ☐
   - MR Angiography ☐
   - CT Angiography ☐

10. Right carotid artery: % stenosis [%] **OR** if artery is completely blocked tick box ➔ ☐

11. Left carotid artery: % stenosis [%] **OR** if artery is completely blocked tick box ➔ ☐

12. Carotid artery scheduled for operation: Right ☐ Left ☐ *(please tick one box)*

RISK FACTORS *(Please tick one box for each line)*

		YES	NO
13.	Hypertension? (on treatment or systolic BP ≥ 160 mmHg or diastolic ≥ 90 mmHg)	☐	☐
14.	Diabetes? (on treatment or any blood sugar ≥ 10 mmol/litre or ≥ 180 mg%)	☐	☐
15.	Peripheral arterial disease?	**YES**	**NO**
15 (a)	- claudication ever?	☐	☐
15 (b)	- previous peripheral arterial surgery?	☐	☐
15 (c)	- previous peripheral angioplasty?	☐	☐

		YES	NO
16.	Aortic aneurysm ever?	☐	☐
17.	Chronic lung disease?	☐	☐
18.	History of angina?	☐	☐
19.	Previous myocardial infarction?	☐	☐
20.	Previous coronary surgery or angioplasty?	☐	☐
21.	Awaiting coronary surgery or angioplasty?	☐	☐
22.	Cardiac failure now or in the past?	☐	☐
23.	Atrial fibrillation now or in the past?	☐	☐
24.	Current smoker (within last 12 months)?	☐	☐
25.	Lifetime non-smoker?	☐	☐

26. Blood pressure
    Systolic I_____________I mmHg
    Diastolic I_____________I mmHg

27. **AMERICAN SOCIETY OF ANAESTHSTISTS CLASSIFICATION** - Physical condition
    ASA GRADE *(please tick one box only)*

I	Normally healthy patient with localised condition requiring surgery	☐
II	Patient with mild or well controlled systemic condition e.g. mild hypertension	☐
III	Patient with severe systemic condition limiting lifestyle e.g. angina	☐
IV	Patient with severe systemic condition threatening life e.g. advanced cardiac disease	☐
V	Moribund patient not expected to survive 24 hours with or without operation	■ **(DO NOT RANDOMISE)**

Either Fax or Telephone your Randomisation:

Fax Randomisation: please fax both pages of this form to the GALA Trial Office, Fax number: **+ 44 (0) 131 332 5150.** The allocation will be faxed back to you within one working day.

Telephone Randomisation (24hour service): telephone **+ 44 (0) 131 537 2922** to randomise your patient.

Once the allocation is received please check one of the boxes below and place this form in the patient's file. Thank you.

Remember: You must fax the Patient's signed and witnessed consent form to the Trial Office + 44 (0) 131 332 5150

FOR GALA TRIAL OFFICE USE ONLY

This patient has been randomised to:

GENERAL ANAESTHESIA ☐ **LOCAL ANAESTHESIA** ☐
