# Supplementary material for: GALA: an international multicentre randomised trial comparing general anaesthesia versus local anaesthesia for carotid surgery
Source: Trials. 2008 May 21;9:28. doi: 10.1186/1745-6215-9-28 (PMC2413207; doi:10.1186/1745-6215-9-28)
Supplement: Additional file 2 — Hospital discharge or 7 day post-surgery follow-up form. The GALA trial post-surgery data collection form. [file 1745-6215-9-28-S2.pdf]

GALA GENERAL ANAESTHESIA vs LOCAL ANAESTHESIA FOR CAROTID SURGERY
HOSPITAL DISCHARGE OR 7 DAY POST-SURGERY FOLLOW-UP FORM

To the surgeon and anesthetist: Please complete questions 1-29 (pages 1, 2 & 3) as soon as you have finished the operation. The other questions should be answered at hospital discharge, or seven days post-surgery or death whichever is soonest. If this patient is no longer going to have surgery please give details of any anaesthetic given and complete questions 1 to 4 (page 1). and 30 to 38 (page 3) together with the contact details (page 4).

HOSPITAL CODE NUMBER: ☐☐☐ or Hospital name |____________________|

PATIENT DETAILS:

Family name: |____________________|

First names: |____________________| Hospital number |____________________|

Date of birth: ____/____/________ *(dd/mm/yyyy)*

SURGERY DETAILS:

1. Was this patient admitted for surgery? **YES** ☐ *Please tick one box*
   NO ☐ ➔ If NO please give reason: ☐

2. Date of admission for surgery *(dd/mm/yyyy)* |____/____/_____|

3. Has the patient taken any of the following in the 48 hours before surgery? *Please tick appropriate box(s)*
   - Aspirin ☐
   - Clopidogrel ☐
   - Dipyridamole ☐
   - Warfarin ☐
   - Other antiplatelet ☐ ➔ Name of drug: ____________________
   - None of the above taken ☐

4. Was carotid surgery carried out? **YES** ☐ *Please tick one box*
   NO ☐
   If NO please give the reason below: (then please answer questions 30 – 38 on p 3 and the contact details on p 4)

   ☐

5. Date of carotid surgery: *(dd/mm/yyyy)* |____/____/______|

6. Side of carotid surgery: **Right** ☐ *Please tick one box*
   Left ☐

7. Type of surgery: **Conventional** ☐ *Please tick one box*
   Eversion ☐
   Other ☐ *If **Other** please say surgery type* |____________________|

8. Duration of surgery:
   (skin to skin time) |____________| **minutes**

		YES	NO	
9.	Shunt used?	☐	☐	If shunt was used please give reason below: ☐

		YES	NO	
10.	Patch used?	☐	☐	
11.	Intraoperative heparin used?	☐	☐	➔ Dose: |_________| units
12.	Heparin reversed?	☐	☐	➔ Dose: |_________| mgs Protamine

13. Weight of patient: |________| pounds **or** |_________| kgs **Actual weight/ Estimated weight** *(delete as appropriate)*

Please turn over/

GENERAL ANAESTHESIA:

Please Note: ONLY answer questions 14 to 18 if this was the principal method of anaesthesia.

		YES	NO
14.	General anaesthesia used?	☐	☐

(If YES complete questions 15 to 18 below)

		YES	NO
15	Pre-medication?	☐	☐
16.	Maintenance technique:		
	- total IV anaesthesia?	☐	☐
	- use of volatile agents?	☐	☐
	- use of nitrous oxide?	☐	☐
	- use of muscle relaxants?	☐	☐
17.	Did blood pressure require manipulation up?	☐	☐
18.	Did blood pressure require manipulation down?	☐	☐

LOCAL ANAESTHESIA:

Please Note: ONLY answer questions 19 to 28 if this was the principal method of anaesthesia.

		YES	NO
19.	Local anaesthesia used?	☐	☐

If YES complete questions 20 to 28 below

		YES	NO
20.	Pre-medication?	☐	☐
21.	Local infiltration used for anaesthesia?	☐	☐
22.	Cervical plexus block used?	☐	☐
23.	Additional intra-operative local anaesthetic?	☐	☐
24.	Did blood pressure require manipulation up?	☐	☐
25.	Did blood pressure require manipulation down?	☐	☐
26.	Intra-operative sedative?	☐	☐
27.	Intra-operative analgesia?	☐	☐
28.	Conversion to general anaesthesia?	☐	☐

FOR ALL PATIENTS:

		YES	NO
29.	Was the type of anaesthesia used the same as allocated at trial entry?	☐	☐

If **NO** please give the reasons in the space below:

____

NAME OF SURGEON CARRYING OUT THE OPERATION |______________________________|

PLEASE INDICATE THE OPERATING SURGEON'S GRADE (tick one box)

Trainee ☐

Consultant ☐ (Consultant = an individual who has completed their training and is capable of doing the procedure with no supervision, and who has an appropriate specialist qualification.)

Other ☐ If you tick OTHER please say what grade below:

|______________________________|

NAME OF MOST SENIOR SURGEON PRESENT AT THE OPERATING TABLE |______________________________|

PLEASE INDICATE THE GRADE OF THE MOST SENIOR SURGEON PRESENT AT THE OPERATING TABLE (tick one box)

Trainee ☐

Consultant ☐ (Consultant = an individual who has completed their training and is capable of doing the procedure with no supervision, and who has an appropriate specialist qualification.)

Other ☐ If you tick OTHER please say what grade below:

|______________________________|

Please turn over/

NAME OF ANAESTHETIST ADMINISTERING ANAESTHESIA |____|

PLEASE INDICATE THE ADMINISTERING ANESTHETIST'S GRADE (tick one box)

Trainee ☐

Consultant ☐ (Consultant = an individual who has completed their training and is capable of doing the procedure with no supervision, and who has an appropriate specialist qualification.)

Other ☐ Please state what grade |____|

NAME OF MOST SENIOR ANAESTHETIST PRESENT IN THE OPERATING THEATRE |____________________________|

PLEASE INDICATE THE GRADE OF THE MOST SENIOR ANAESTHETIST PRESENT IN THE OPERATING THEATRE (tick one box)

Trainee ☐

Consultant ☐ (Consultant = an individual who has completed their training and is capable of doing the procedure with no supervision, and who has an appropriate specialist qualification.)

Other ☐ Please state what grade |____|

Please complete the rest of this form
at hospital discharge, or 7 days post-surgery or death, whichever occurs first.

COMPLICATIONS:
Since randomisation has the patient had any of the following? *(Please tick all that apply)*

		YES	NO	For any YES answers please give the date below:
30.	Stroke of any type (> 24 hours)? *	☐	☐	|_____/_____/_______| (dd/mm/yyyy)
31.	Transient ischaemic attack (brain) (< 24 hours)? *	☐	☐	|_____/_____/_______| (dd/mm/yyyy)
32.	Retinal infarction (> 24 hours)? *	☐	☐	|_____/_____/_______| (dd/mm/yyyy)
33.	Amaurosis fugax (< 24 hours)? *	☐	☐	|_____/_____/_______| (dd/mm/yyyy)
34.	Myocardial infarction? *	☐	☐	|_____/_____/_______| (dd/mm/yyyy)
35.	New or worsening angina?	☐	☐	|_____/_____/_______| (dd/mm/yyyy)
36.	New arrhythmia requiring treatment?	☐	☐	|_____/_____/_______| (dd/mm/yyyy)
37.	New or worsening heart failure?	☐	☐	|_____/_____/_______| (dd/mm/yyyy)
38.	Has this patient died? *	☐	☐	|_____/_____/_______| (dd/mm/yyyy)

If this patient has died please give cause of death below:

Cause of death:

* **If you have answered Yes to any question above with an asterisk (*) please complete a MAJOR EVENT FORM and send it to the GALA Trial Office**

Between the induction of anaesthesia and today's date did the patient have any of the following?

		YES	NO
39.	Deep vein thrombosis?	☐	☐
40.	Pulmonary embolism?	☐	☐
41.	Retention of urine?	☐	☐
42.	Chest infection?	☐	☐
43.	Wound haematoma?	☐	☐
44.	Wound infection?	☐	☐
45.	Any other medical or surgical complication?	☐	☐

Q45 - If YES please describe below ➘

46. Did this patient require re-operation? **YES** ☐ **NO** ☐

If **YES** please give the reason ➘

Please turn over/

PLACE AND DURATION OF HOSPITAL STAY:

47.	Number of hours spent in recovery room after surgery	[ ]	hours	
48.	Number of hours spent in Intensive Therapy Unit after surgery (Level 3)	[ ]	hours	Level 3 - Intensive Care. Nurse patient ratio 1:1, with dedicated senior and trainee medical staff.
49.	Number of hours spent in High Dependency Unit after surgery (Level 2, Level 1)	[ ]	hours	Level 2 - High Dependency with organ support, but not mechanical ventilation. Nurse patient ratio 1:2, with dedicated senior and trainee medical staff.<br>Level 1 - HDU. Close monitoring. Nurse patient ratio 1:4. No dedicated medical staff.

50. Has this patient been discharged from hospital? **YES** [ ] **NO** [ ]

51. If YES give Date of discharge *(dd/mm/yyyy)* |_____/_____/______|

OR

If still in hospital, give Ward number or name: Ward |____|

If still in hospital, give the name of the doctor responsible for their care Dr |____|

CONTACT DETAILS: PATIENT'S FULL POSTAL ADDRESS

(Please print clearly or attach a sticky label)

Post code	
Telephone	

PATIENT'S FAMILY DOCTOR DETAILS

Family Doctor's name	
Family Doctor's full postal address	
Post code	
Telephone	

NAME OF COLLABORATOR COMPLETING THIS FORM |____|

TODAY'S DATE |___/___/______| dd/mm/yyyy

Please post or fax this form to:

GALA Trial Co-ordinator, Neurosciences Trials Unit, Bramwell Dott Building,
Western General Hospital, Crewe Road, Edinburgh EH4 2XU.
Fax: + 44 131 332 5150
(an envelope is provided)
