# Supplementary material for: GALA: an international multicentre randomised trial comparing general anaesthesia versus local anaesthesia for carotid surgery
Source: Trials. 2008 May 21;9:28. doi: 10.1186/1745-6215-9-28 (PMC2413207; doi:10.1186/1745-6215-9-28)
Supplement: Additional file 3 — One month post-surgery follow-up form. The GALA trial one-month post-surgery data collection form, for use at examination by independent neurologist. [file 1745-6215-9-28-S3.pdf]

# GALA GENERAL ANAESTHESIA vs LOCAL ANAESTHESIA FOR CAROTID SURGERY
## ONE MONTH POST-SURGERY FOLLOW-UP FORM

## To the physician: Please complete this form for your patient at their follow-up appointment 30 DAYS after their carotid surgery

**HOSPITAL CODE NUMBER** ☐☐☐ |______________________________|
Hospital name if code not available

**PATIENT DETAILS:**

Family name: |______________________________|

First names: |____________________| Hospital number |____________|

Date of birth: dd |____| / mm |____| / yyyy |______|

**DISCHARGE DETAILS**

1. Has this patient been discharged from hospital? **YES** ☐ **NO** ☐ *(Please tick one box)*

If **YES** give Date of discharge *(dd/mm/yyyy)* |____/____/_____|

**OR**
If still in hospital, give Ward number or name: Ward |______________________|

If still in hospital, give the name of the doctor responsible for their care Dr |______________________|

2. Did the patient require **re**-operation? **YES** ☐ **NO** ☐

*If **YES** please give the reason below:*

☐

**COMPLICATIONS**

**Between randomisation and today's appointment date (including the pre-, peri-, and post-operative periods) did this patient have any of the following?** *(Please answer Yes or No for each question)*

| | | YES | NO | | For any YES answers please give the date below: |
|---|---|---|---|---|---|
| 3. | Stroke of any type (more than 24 hours)? * | ☐ | ☐ | → | 3. \|___/___/_____\| (dd/mm/yyyy) |
| 4. | Transient ischaemic attack (brain) (less than 24 hours)? * | ☐ | ☐ | → | 4. \|___/___/_____\| (dd/mm/yyyy) |
| 5. | Retinal infarction (more than 24 hours)? * | ☐ | ☐ | → | 5. \|___/___/_____\| (dd/mm/yyyy) |
| 6. | Amaurosis fugax (less than 24 hours)? * | ☐ | ☐ | → | 6. \|___/___/_____\| (dd/mm/yyyy) |
| 7. | Myocardial infarction? * | ☐ | ☐ | → | 7. \|___/___/_____\| (dd/mm/yyyy) |
| 8. | New or worsening angina? | ☐ | ☐ | → | 8. \|___/___/_____\| (dd/mm/yyyy) |
| 9. | New arrhythmia requiring treatment? | ☐ | ☐ | → | 9. \|___/___/_____\| (dd/mm/yyyy) |
| 10. | New or worsening heart failure? | ☐ | ☐ | → | 10.\|___/___/_____\| (dd/mm/yyyy) |
| 11. | Has this patient died? * | ☐ | ☐ | → | 11.\|___/___/_____\| (dd/mm/yyyy) |

**If this patient has died please give cause of death below:**

☐

* **If you have answered Yes to any question above with an asterisk (*) please complete a MAJOR EVENT FORM and send it to the GALA Trial Office**

**Please turn over/**

**Between the induction of anaesthesia and today's appointment date did the patient have any of the following?**

| | *(Please answer Yes or No for each question)* | YES | NO |
|---|---|---|---|
| 12. | Deep vein thrombosis? | | |
| 13. | Pulmonary embolism? | | |
| 14. | Retention of urine? | | |
| 15. | Chest infection? | | |
| 16. | Wound haematoma? | | |
| 17. | Wound infection? | | |
| 18. | Headache ipsilateral to surgery? | | |
| 19. | Lower cranial nerve injury (weak face or tongue, hoarseness etc.)? | | |

*Q19 - If **YES** please describe below*

| | | YES | NO |
|---|---|---|---|
| 20. | Any other medical or surgical complication? | | |

*Q20 - If **YES** please describe below*

**NAME OF INDEPENDENT STROKE PHYSICIAN OR NEUROLOGIST COMPLETING THIS FORM:** |__________________________________________|

**TODAY'S APPOINTMENT DATE:** (dd/mm/yyyy) |_____/_____/_______|

**Please post or fax this form to:**

GALA Trial Co-ordinator, Neurosciences Trials Unit, Bramwell Dott Building,
Western General Hospital, Edinburgh EH4 2XU.
Fax: +44 131 332 5150
(an envelope is provided)
